# Supplementary material for: Mosquito community composition shapes virus prevalence patterns along anthropogenic disturbance gradients
Source: eLife. 2023 Sep 13;12:e66550. doi: 10.7554/eLife.66550 (PMC10547478; doi:10.7554/eLife.66550)
Supplement: Supplementary file 2. — No significant differences were detected for FERV (n = 20), JONV (n = 17), Spilikins virus (n = 12), and Cimo flavivirus I (n = 13; no findings in camp and village). Combinations with zero virus detections shown in light gray. All virus detections were positively associated with the number of mosquitoes per pool (model estimates not shown). [file elife-66550-supp2.docx]

SI Table 2

**Estimate Std. Error z value Pr(>|z|)**

Gouleako virus GOLV (n =33; mainly found in *Culex nebulosus*)

Secondary_forest - Primary_forest == 0 0.5280 0.8825 0.598 0.9743

Agriculture - Primary_forest == 0 0.6876 0.8567 0.803 0.9271

Camp - Primary_forest == 0 1.5425 0.8368 1.843 0.3393

**Village - Primary_forest == 0 2.2850 0.7857 2.908 0.0287 ****

Agriculture - Secondary_forest == 0 0.1597 0.6912 0.231 0.9993

Camp - Secondary_forest == 0 1.0145 0.6728 1.508 0.5475

**Village - Secondary_forest == 0 1.7570 0.5976 2.940 0.0258 ****

Camp - Agriculture == 0 0.8549 0.6387 1.338 0.6584

**Village - Agriculture == 0 1.5974 0.5531 2.888 0.0302 ****

Village - Camp == 0 0.7425 0.5398 1.375 0.6345

Herbert virus HEBV (n = 42, mainly found in *Culex nebulosus*)

Secondary_forest - Primary_forest == 0 0.87511 0.69710 1.255 0.7129

Agriculture - Primary_forest == 0 0.81205 0.69924 1.161 0.7684

Camp - Primary_forest == 0 1.12481 0.72942 1.542 0.5278

**Village - Primary_forest == 0 2.00241 0.66530 3.010 0.0213 ****

Agriculture - Secondary_forest == 0 -0.06306 0.52585 -0.120 1.0000

Camp - Secondary_forest == 0 0.24969 0.57081 0.437 0.9922

Village - Secondary_forest == 0 1.12730 0.47560 2.370 0.1199

Camp - Agriculture == 0 0.31275 0.57330 0.546 0.9820

***Village - Agriculture == 0 1.19036 0.47411 2.511 0.0853* ***

Village - Camp == 0 0.87761 0.53111 1.652 0.4566

Cimo Rhabdovirus (n = 40; no findings in primary forest, mainly found in *Culex decens*)

Secondary_forest - Primary_forest == 0 17.9405 1039.1013 0.017 1.00000

Agriculture - Primary_forest == 0 17.4639 1039.1013 0.017 1.00000

Camp - Primary_forest == 0 16.4038 1039.1014 0.016 1.00000

Village - Primary_forest == 0 14.8373 1039.1016 0.014 1.00000

Agriculture - Secondary_forest == 0 -0.4766 0.3952 -1.206 0.69378

***Camp - Secondary_forest == 0 -1.5368 0.6547 -2.347 0.09673* ***

**Village - Secondary_forest == 0 -3.1033 0.8911 -3.482 0.00295 *****

Camp - Agriculture == 0 -1.0602 0.6694 -1.584 0.43874

**Village - Agriculture == 0 -2.6267 0.8904 -2.950 0.01802 ****

Village - Camp == 0 -1.5665 1.0450 -1.499 0.49468

Cavally virus CAVV (n = 30; mainly found in *Culex nebulosus*)

Secondary_forest - Primary_forest == 0 0.329395 0.750648 0.439 0.9921

Agriculture - Primary_forest == 0 0.005789 0.787960 0.007 1.0000

Camp - Primary_forest == 0 0.367899 0.837351 0.439 0.9920

***Village - Primary_forest == 0 1.737325 0.677846 2.563 0.0751* ***

Agriculture - Secondary_forest == 0 -0.323606 0.692784 -0.467 0.9899

Camp - Secondary_forest == 0 0.038504 0.754410 0.051 1.0000

***Village - Secondary_forest == 0 1.407930 0.560918 2.510 0.0857* ***

Camp - Agriculture == 0 0.362110 0.791146 0.458 0.9907

**Village - Agriculture == 0 1.731536 0.602387 2.874 0.0318 ****

Village - Camp == 0 1.369426 0.681085 2.011 0.2549

Cimo-phenuivirus II (no findings in village and agriculture, mainly found in *Uranotaenia spec.*)

***Secondary_forest - Primary_forest == 0 -2.6351 1.0884 -2.421 0.0757* ***

Agriculture - Primary_forest == 0 -20.0109 2613.6108 -0.008 1.0000

Camp - Primary_forest == 0 -0.7653 0.7267 -1.053 0.7803

Village - Primary_forest == 0 -21.4366 2862.1448 -0.007 1.0000

Agriculture - Secondary_forest == 0 -17.3758 2613.6109 -0.007 1.0000

Camp - Secondary_forest == 0 1.8698 1.1815 1.583 0.4277

Village - Secondary_forest == 0 -18.8015 2862.1449 -0.007 1.0000

Camp - Agriculture == 0 19.2456 2613.6108 0.007 1.0000

Village - Agriculture == 0 -1.4257 3875.9298 0.000 1.0000

Village - Camp == 0 -20.6713 2862.1448 -0.007 1.0000
