# Supplementary material for: Mosquito community composition shapes virus prevalence patterns along anthropogenic disturbance gradients
Source: eLife. 2023 Sep 13;12:e66550. doi: 10.7554/eLife.66550 (PMC10547478; doi:10.7554/eLife.66550)
Supplement: Supplementary file 3. [file elife-66550-supp3.docx]

**Supplementary File 2:** Primer pairs used for generic RT-PCR assays.

| Virus taxon | Primer - First round | | Primer – Nested | |
| --- | --- | --- | --- | --- |
|  | Forward | Reverse | Forward | Reverse |
| *Peribunyaviridae ^1^* | 5’-CAAARAACAGCAAAAGAYAGRGARA-3’ | 5’-TTCAAATTCCCYTGiARCCARTT-3’ | 5’-ATGATTAGYAGRCCDGGHGA-3’ | 5’-CTTGACATRTCWGCATTDATYTC-3’ |
| *Jonviridae* | 5’-CACAACCAGKAARCAiCA-3’ | 5’-AGCATGGAATGARTCRTC-3’ | 5’-CACAACCAGKAARCAiCA-3’ | 5’-GAAGTTAAGCWTiCCYTG-3’ |
| *Feraviridae* | 5’-CATGTCGGAKAARGAiCA-3’ | 5’-AACAGGGAAGGARTCRTC-3’ | 5’-CATGTCGGAKAARGAiCA-3’ | 5’-CTTATGTTTAACWTiCCYTG-3’ |
| *Rhabdoviridae* | 5’-GACTACGAiAARTGGAA-3’ | 5’-AGCATYTGRTTRTCiCC-3’ | 5’-GACTACGAiAARTGGAA-3’ | 5’-ACACTCCAiCCYTTYTG-3’ |
| *Flavivirus ^2^* | 5’-CATTTGGTACATGTGGYT-3’ | 5’-CACAACACMRTCRTCiCC-3’,  5’-CACCGCYRMATCATCiCC-3’ * | 5’-CGTAGCWGGMTGGGAYAC-3’ | 5’-CTGTCCTGAiCCTCKYTG-3’,  5’-TGAGCCACTiCCWCKYTG-3' * |
| *Iflavirus* | 5’-TCAGTAGATTGTYTiAARGAT-3’ | 5’-GACATTAGTACRTCRTCiCC-3’ | 5’-GATTATTCGAAiTTYGGiCC-3’ | 5’-GACATTAGTACRTCRTCiCC-3’ |
| *Orbivirus* | 5’-TTCTGGATWTGGiGARGG-3’ | 5’-AACGTATCRTCiCCMAC-3’ | 5’-TGGAACATTRTGGARYGG-3’ | 5’-TGCAAGTGTiGARTTYTC-3’ |
| Cimodo virus | 5’-ATTGCRTCSGGAAAACAGACCG-3’ | 5’-GTCATAGCGTTATCTGCAGCCTC-3’,  5’-GTCATGGCATTATCCACAGCTTC-3’ * | 5’-TGTCCGCGATATGTTAAAAGTGTT-3’,  5’-TGTCCGTGACATGCTTAAAGTGTT-3’ * | 5’-GGCCTTTGGTTAAATGTTGCCG-3’,  5’-TCAGATCGTTGATTAAATGTAGCCG-3’ * |

^1^ Additional semi-nested PCRs using either the forward or the reverse primer of the first round and the corresponding nested primer were performed.

^2^Additional semi-nested PCR using the reverse primer of the first round and the forward primer of the nested round was performed.

* Primers were mixed 1:1.
